# The effect of CRM1 inhibition on human non-Hodgkin lymphoma cells

**DOI:** 10.1038/s41408-019-0188-6

**Published:** 2019-02-26

**Authors:** Jithma P. Abeykoon, Jonas Paludo, Kevin E. Nowakowski, Mary J. Stenson, Rebecca L. King, Linda E. Wellik, Xiaosheng Wu, Thomas E. Witzig

**Affiliations:** 1 Division of Hematology, Department of Internal Medicine, Mayo Clinic, Rochester, MN United States; 2 Department of Laboratory Medicine and Pathology, Mayo Clinic, Rochester, MN United States

Malignant cells, including lymphoma cells, have an increased dependence on nuclear-cytoplasmic trafficking of protein and RNA molecules compared to normal cells due to their high metabolic demand and proliferative potential^1,2^. Chromosome region maintenance 1 (CRM1, also known as Exportin-1 or XPO1), the most prominent protein export receptor, recognizes leucine-rich nuclear export sequences of cargo proteins and is necessary for the nuclear export of many proteins, including tumor suppressor proteins (TSPs)^1,2^. The overexpression of CRM1 protein observed in both solid and hematologic malignancies led to the hypothesis that CRM1 is an important promoter or sustainer of cancer cell survival through the enhanced transportation of TSPs out of the nucleus where they normally function^2,3^. KPT-330 (Selinexor, Karyopharm, Newton, MA) is an oral inhibitor of CRM1 which is currently in phase I and II clinical trials that are evaluating its activity in patients with relapsed and/or refractory diffuse large B-cell lymphoma (DLBCL).

Pre-clinical studies assessing the effect of KPT-330 in mantle cell lymphoma (MCL) and T-cell lymphoma (TCL) remain scarce and patients with MCL and TCL have not yet been the focus of clinical trials of KPT-330. There remains an unmet need for an effective new treatment for MCL and TCL, especially with agents that offer a new mechanism of action. Therefore, with special emphasis on MCL and TCL, we designed this pre-clinical study to evaluate the ex vivo effect of CRM1 inhibition using KPT-330 as monotherapy and in combination therapy in MCL, TCL, and DLBCL human cell lines with the goal to provide a rationale for potential clinical trials using CRM1 inhibitors in these diseases.

The expression of CRM1 was analyzed via immunoblotting and immunohistochemistry. Cellular proliferation and cell cycle analysis were assessed through ^3^[H] thymidine labeling, and viability was assessed through annexin V and propidium iodide (PI) labeling. Immunofluorescence microscopy was performed to localize i-kappa–beta (IkB) before and after drug treatment. A combination index (CI)<1 was considered to be synergistic. A detailed explanation of the methods used is included in the supplementary material.

We first examined the expression of CRM1 in DLBCL, TCL, and MCL cell lines through immunoblotting. CRM1 was overexpressed in non-Hodgkin lymphoma (NHL) cells (i.e. DLBCL, TCL, and MCL) when compared to normal blood and tonsillar B-cells and T-cells (Fig. 1a). Moreover, Supplemental Fig. 1 illustrates a representative depiction of CRM1 expression by immunohistochemistry in patient samples of DLBCL, TCL, and MCL. Given its overexpression in lymphoma cells, we then questioned if CRM1 is targetable in these NHL types using the new CRM1 inhibitor, KPT-330. We assessed the anti-proliferative effect of KPT-330 on TCL (Karpas-299 and SR-786), MCL (JVM-2 and Jeko-1), and DLBCL (LY-1 and DHL-2) by ^3^H-thymidine incorporation. KPT-330 was markedly anti-proliferative in TCL and MCL cell lines in concentrations as low as 100 ηM, whereas the inhibition of proliferation by KPT-330 was less pronounced in DLBCL cell lines and required concentrations as high as 0.5 µM (Fig. 1b).

**Fig. 1:**
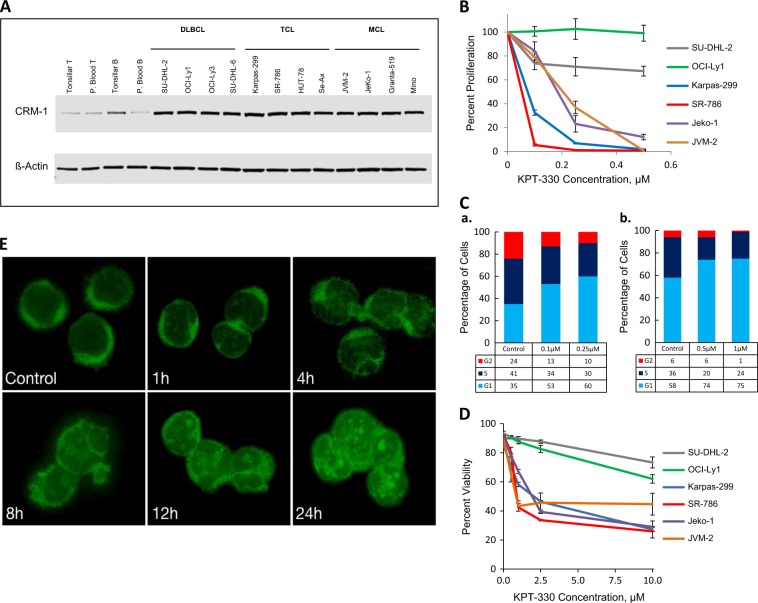
**Expression and targeting of CRM1 in non-Hodgkin lymphoma cells**. **a** Immunoblot showing the expression of CRM1 protein in DLBCL, TCL, and MCL cell lines, and normal B and T-cells. **b** Proliferation assay on representative TCL, MCL, and DLBCL cell lines treated with various concentrations of KPT-330 for 48 h. Data shown in percentage are normalized with non-drug treated controls. **c** Cell cycle analysis of representative cell lines (**a**) Karpas-299 (TCL) and (**b**) Jeko-1 (MCL) after 24 h incubation with various concentrations of KPT-330. **d** Apoptosis assay data on representative cell lines of TCL, MCL, and DLBCL after treatment with various concentrations of KPT-330 for 48 h. **e** Immunofluorescence staining of IkB-alpha in Jeko-1 (MCL) cells upon treatment with 2.5 µM of KPT-330 for 24 h. *DLBCL* diffuse large B-cell lymphoma, *MCL* Mantle cell lymphoma, *TCL* T-cell lymphoma, *CRM1* chromosome region maintenance 1

The strong anti-proliferative effect of KPT-330 on MCL and TCL cell lines suggested that KPT-330 may affect the cell cycle of MCL and TCL cells. To that end, we evaluated the potential impact on the cell cycle using flow cytometry and detected an increase in the G1 fraction when Jeko-1 and Karpas-299 cell lines were treated with KPT-330 (Fig. 1c), suggesting that KPT-330 induces cell cycle arrest at the G1 phase in MCL and TCL, respectively. KPT-330 had no such effect on the cell cycle of DLBCL cell lines (LY-1 and DHL-2) at concentrations ranging from 0.5 µM to 10 µM (data not shown). Following the observations that CRM1 inhibition induces an anti-proliferative effect through G1 cell cycle arrest, we next assessed the impact on cellular viability. As shown in Fig. 1d, CRM1 inhibition with KPT-330 effectively induced cell apoptosis in a dose-dependent fashion in all TCL and MCL cell lines tested. The DLBCL cell lines also responded to treatment but, as before, required higher concentrations of KPT-330 to induce the anti-tumor effect (Fig. 1d).

Given its anti-tumor activity as a single agent in NHL cells, we hypothesized that the combination of KPT-330 with other agents may further enhance the anti-tumor activity. Bortezomib is a proteasome inhibitor with effects on the NF-kB pathway.^4^ It has been shown to have synergy with KPT-330 in multiple myeloma (MM) and is approved for the treatment of relapsed MCL.^5^ Considering that genotoxic stress could enhance cellular dependency on nuclear cytoplasmic export, we also tested gemcitabine, a drug that is incorporated into DNA and interferes with DNA synthesis, and is used widely as a salvage therapy for relapsed NHL.^6^ KPT-330 together with gemcitabine or bortezomib exhibited moderate to strong synergistic effects on cell proliferation in TCL and MCL cell lines as revealed by the CI scores of <1 (Table 1 and Supplemental Figure 2). No synergistic effect was observed in DLBCL cell lines when KPT-330 at concentrations ranging from 0.1 μM to 1 μM were combined with bortezomib at concentrations ranging from 2 ηM to 10 ηM, or gemcitabine at concentrations ranging from 1 ηM to 50 ηM (data not shown).

**Table 1:** The synergistic antitumor effect of KPT-330 combined with bortezomib or gemcitabine in TCL and MCL cell lines. Synergy was defined as a combination index<1

		Gemcitabine (ηM)					Bortezomib (ηM)		
		1	10	20	50		2	5	10
TCL	KPT 0.1 µM (CI)	3.2	1.5	0.67	0.96	KPT 0.1 µM (CI)	1.37	0.88	0.54
	KPT 0.25 µM (CI)	1.82	1.8	0.87	0.78	KPT 0.25 µM (CI)	1.11	0.84	0.46
	KPT 0.5 µM (CI)	0.88	1.33	0.60	0.55	KPT 0.5 µM (CI)	0.65	0.40	0.25
MCL	KPT 0.1 µM (CI)	2.36	0.59	0.69	1.06	KPT 0.1 µM (CI)	0.71	0.61	0.39
	KPT 0.25 µM (CI)	1.97	0.66	0.65	1.01	KPT 0.25 µM (CI)	1.41	1.04	0.48
	KPT 0.5 µM (CI)	0.70	0.61	0.65	0.94	KPT 0.5 µM (CI)	0.75	0.63	0.14

*TCL* T-cell lymphoma (representative cell line: SR-786), *MCL* mantle cell lymphoma (representative cell line: JVM-2), *CI* combination index, *KPT* KPT-330

Constitutive activation of NF-kB pathways is critical for the proliferation and survival of many lymphoma cell lines and previous studies have shown that KPT-330 acts through NF-kB deactivation^7,8^. Hence, to better understand the mechanism of KPT-330 mediated lymphoma cell killing, we hypothesized that nucleocytoplasmic shuttling of proteins involved in the NF-kB pathway may be affected by KPT-330, leading to the inactivation of NF-kB signaling. Therefore, we examined the subcellular localization of the tumor suppressor IkB by immunofluorescence staining in untreated and KPT-330 treated cells. As shown in Fig. 1e, in untreated cells IkB was mainly localized in the cytoplasmic compartment, whereas IkB progressively accumulated in the nucleus following KPT-330 treatment over time. Our data further suggests that one result of CRM1 inhibition is the disruption of the nuclear efflux of IkB necessary for the lymphoma cells to function.

In this study, we analyzed the antitumor effects of CRM1 inhibition with KPT-330 in NHL cell lines with a focus on TCL and MCL. We demonstrated that NHL cells indeed overexpress CRM1, and the CRM1 inhibitor KPT-330 has potent anti-proliferative and pro-apoptotic effects on TCL and MCL cell lines. Consistent with previous studies of KPT-330 on acute myeloid leukemia, our studies of KPT-330 in NHL showed induction of G1 cell cycle arrest in MCL and TCL^9^. As was shown in sarcoma and MCL in previous studies, we also found that MCL cells have increased IkB localization in the nucleus with KPT-330 treatment^10,11^. In recent studies, it was found that bortezomib and DNA damaging agents such as gemcitabine impose a synergistic antitumor effect when combined with KPT-330 in solid tumors such as sarcoma and breast cancer, and hematologic malignancies such as AML, MM, and DLBCL^4–6,8,12^. Paralleling these findings, the synergistic antitumor effect when KPT-330 was combined with bortezomib or gemcitabine in TCL and MCL found in our study suggests that combining KPT-330 with these agents may increase the overall anti-tumor effect and should be further explored. In summary, CRM1 inhibition via KPT-330 has the potential to become a new therapeutic option in the treatment of TCL and MCL and deserves clinical investigation in these patient populations as a single-agent or in combination with other active drugs.

## Supplementary information

Supplementary Material.

Supplemental Figure 1

Supplemental Figure 2
