# Supplementary material for: The effect of CRM1 inhibition on human non-Hodgkin lymphoma cells
Source: Blood Cancer J. 2019 Feb 26;9(3):24. doi: 10.1038/s41408-019-0188-6 (PMC6391437; doi:10.1038/s41408-019-0188-6)
Supplement: Supplementary file 1 — Supplementary Material. [file 41408_2019_188_MOESM1_ESM.docx]

**The Effect of CRM1 Inhibition on Human Non-Hodgkin Lymphoma Cells**

**Supplementary material**

1. Methods

*Cell lines*

All lymphoma cell lines used in this study were purchased from the cell line repositories ATCC (Manassas, VA) or DSMZ (Braunschweig, Germany). These included MCL cell lines: Jeko-1, Mino, JVM-2 and Granta-519; TCL cell lines: Karpas-299, SR-786, Se-Ax and HUT-78; and DLBCL cell lines: OCI-Ly1 (LY-1), SU-DHL-2 (DHL-2), OCI-Ly3 (LY-3) and SU-DHL-6 (DHL-6). TCL and MCL cell lines were cultured in RPMI-1640 supplemented with 10% fetal bovine serum, while DLBCL cell lines were maintained in IMDM with 10% human serum (Sigma). Cells used in experiments had viability counts of 85% or greater before treatment.

*Reagents and drugs*

Protease inhibitor was purchased from Sigma-Aldrich, St. Louis, MO, reference number: 11836153001. Phenylmethanesulfonyl fluoride (PMSF) was purchased from Sigma-Aldrich, catalog number: 93482, and HALT phosphatase inhibitor was purchased through Thermo Fisher Scientific, Waltham, MA, product number: 1862495. Beta-mercaptoethanol was purchased from Sigma-Aldrich, CAS number: 60-24-2. ^3^[H] labeled thymidine was purchased from Perkin Elmer, Waltham, MA. Annexin V was purchased through Invitrogen, Life Technologies, Carlsbad, CA and propidium iodide was purchased through Sigma-Aldrich, CAS number: 25535-16-4. Bortezomib was purchased from Selleckchem, Houston, TX; catalog No: S1013 and gemcitabine was purchased through Sigma-Aldrich, CAS number: 122111-03-09. KPT-330 was purchased from Selleckchem; catalog No: S7252. Dimethyl sulfoxide was purchased from Sigma-Aldrich, catalog number: D650. Phosphate buffer solution (PBS) was purchased through Life Technologies.

*Immunoblot*

Lymphoma and normal control cells were extracted in lysis buffer containing protease inhibitor, PMSF, and HALT phosphatase inhibitor for total cellular proteins. The cell lysates were diluted in Laemmli sample buffer supplemented with beta-mercaptoethanol. The proteins were resolved in precast 4-15% gradient Criterion TGX Midi protein gels (Bio-Rad) by electrophoresis, and then transferred to PVDF membranes. The membranes were blocked with 1:1 LI-COR ODB/PBS buffer and probed with rabbit anti-human CRM1 primary antibody (Cell Signaling, Danvers, MA; catalog number: 46249) and mouse anti-human beta-actin antibody (Santa Cruz Biotechnology, Dallas, TX; catalog number: SC-47778), followed with fluorescent secondary antibodies; anti-rabbit IRDye 800CW or anti-mouse IRDye 700 CW (LI-COR) for one hour. The membranes were imaged on a LI-COR Odyssey CLX imager.

*Treatment for proliferation and apoptosis assays*

To assess cellular proliferation, cells were treated with KPT-330 at 0.0µM, 0.1µM, 0.25µM and 0.5µM concentrations, and incubated for 48 hours. Dimethyl sulfoxide was used to substitute the drug in controls in the proliferation and apoptosis assays. For apoptosis studies, all cell lines were treated with KPT-330 at 0.0µM, 0.5µM, 1.0µM, 2.5µM and 10µM concentrations for 48 hours prior to analysis and all experiments were done in triplicates.

*Proliferation assay*

Cells were seeded in a 96 well plate with 0.0µM, 0.1µM, 0.25µM, and 0.5µM KPT-330. After 48 hours of incubation, ^3^[H] labeled thymidine (CMP) was added. The plate was harvested after another 18 hours of incubation. ^3^[H]-thymidine uptake was measured on a MicroBeta workstation (Perkin Elmer). All experiments were performed in triplicates.

*Cell cycle analysis*

For cell cycle analysis, the Karpas-299 cell line was incubated with 0.0µM, 0.1µM, and 0.25µM of KPT-330; Jeko-1 cell line was incubated with 0.0µM, 0.5µM and 1.0µM concentrations of KPT-330; and, the LY-1 and DHL-2 cell lines were incubated at 0.0µM, 0.5µM, 1.0µM, 2.5µM and 10µM of KPT-330 for 24 hours. Dimethyl sulfoxide was used to substitute the drug in controls. Following incubation, cells were fixed with 70% cold ethanol and kept at 4C^o^ for 24 hours, followed by PI staining. Cell cycle analysis was done by using a BD FACS Caliber flow cytometer (BD Biosciences) and analyzed using ModFit^®^ Software.

*Apoptosis assay*

After incubation, cells were washed with Annexin buffer and stained with both propidium iodide (PI) and FITC-Annexin V, and assayed on a BD FACS Caliber flow cytometer (BD Biosciences). Apoptosis results were analyzed with FlowJo software (Tree Star).

*Drug combination treatment*

KPT-330 was used in combination with gemcitabine or bortezomib on cell lines SR-786, JVM-2, LY-1 and DHL-2 for a 48 hour treatment. When gemcitabine was combined with KPT-330, concentrations of 1ηM, 10ηM, 20ηM and 50ηM of gemcitabine were used with 0.1µM, 0.25µM and 0.5µM concentrations of KPT-330. When bortezomib was combined with KPT-330, concentrations of 2ηM, 5ηM and 10ηM of bortezomib were used with 0.1µM, 0.25µM and 0.5µM concentrations of KPT-330. Results from the proliferation assay were used to assess potential synergistic effects and CalcuSyn software was used to mathematically assess the synergistic effect. The combination index <1 was considered to be synergistic. (13)

*Immunofluorescence microscopy*

Jeko-1 cells were treated with KPT-330 at 0µM and 2.5µM concentrations and incubated for 1, 4, 8, 12 and 24 hours. Subsequently, approximately 50,000 cells were plated accordingly on a slide using Cytospin. Cells were fixed in 4% paraformaldehyde in PBS and permeabilized with 0.2% Triton X-100 in PBS for 10 min. Following permeabilization, the cells were incubated overnight with the primary rabbit anti-I-kappa-Beta-alpha [Abcam (ab32518)] diluted in Von Gogh diluent at 1:50, followed by 1-hour incubation with the secondary anti-rabbit antibody conjugated with FITC at a dilution of 1:500 with the Von Gogh diluent. Cells were then analyzed using florescent microscopy after mounting coverslips in Vectastain (Vector Laboratories) containing DAPI (4′, 6′- diamido-2-phenylindole). Fluorescent microscopy was performed with a ZEISS Axiocam MRm with 650x magnification.

*Immunohistochemical staining of CRM1*

Patients’ tissue samples were obtained after the approval of Mayo Clinic Institutional Review Board and immunohistochemical staining of CRM1 on primary lymphoma tissues were done according to standard protocol. Four microns sections of paraffin block were mounted on charged slides and the slides were oven dried for 60 minutes in 60°C. Subsequently, the slides were de-paraffinized in Xylene for 5 minutes and placed in 1% iodine/xylene for 5 minutes. Then the slides were rinsed in clean xylene and passed through 95% ethanol. Following this preparation, the slides were blocked for endogenous peroxidase with a one-to-one solution of 3% hydrogen peroxide and absolute methanol for 10 minutes, and were rinsed well in running tap water. Subsequently, heat-induced epitope retrieval was done through steaming with Citrate pH of 6.1 for 30 minutes and left 5 minutes for cool down. After rinsing in running tap water, the slides were stained the following order; (1) Exportin-1/CRM1(Cell Signaling Technology) 1/200 dilution, (2) MACH 3 ™ Rabbit Probe HRP Polymer Kit (Biocare Medical, Walnut Creek, CA), (3) R-Polymer HRP: MACH 3™ Rabbit Probe HRP Polymer Kit (Biocare Medical, Walnut Creek, CA), (4) Chromogen: DAB+ (DakoCytomation, Carpinteria, CA). Each staining was performed with two 5 minute incubations with water rinse in between. Hemotoxylin was used as the counter stain.

2. Supplementary Figure 1: Immunohistochemistry of CRM1 expression in formalin-fixed, paraffin-embedded lymphoma samples from patients.

A. CRM1 expression in diffuse large B-cell lymphoma. B. CRM1 expression in anaplastic lymphoma kinase negative anaplastic T-cell lymphoma. C. CRM1 expression in mantle cell lymphoma (blastoid variant).

3. Supplementary Figure 2: Synergistic antitumor effect following KPT-330 combination with bortezomib or gemcitabine.

A. Percent proliferation of TCL (SR-786) and, B. MCL (JVM-2) cell line, when treated with KPT-330 +/- gemcitabine. C. Percent proliferation of TCL (SR-786) and, D. MCL (JVM-2) cell line when treated with KPT-330 +/- bortezomib. The proliferative potential has been normalized based on the proliferative potential of the control for each representative cell line. KPT: KPT-330; Gem: Gemcitabine; Bortz: Bortezomib
