# Supplementary figures and images for: The effect of CRM1 inhibition on human non-Hodgkin lymphoma cells
Source: Blood Cancer J. 2019 Feb 26;9(3):24. doi: 10.1038/s41408-019-0188-6 (PMC6391437; doi:10.1038/s41408-019-0188-6)

A

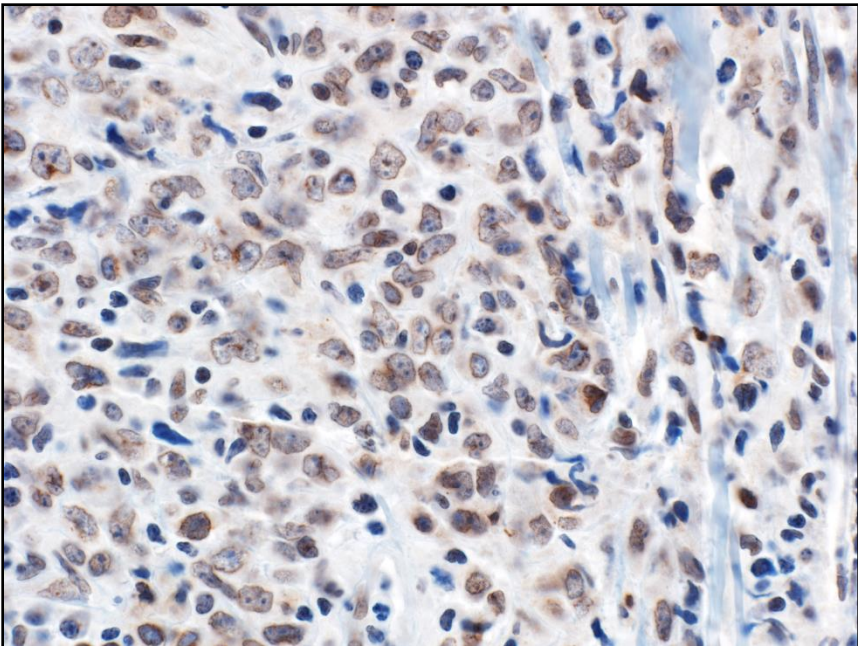

B

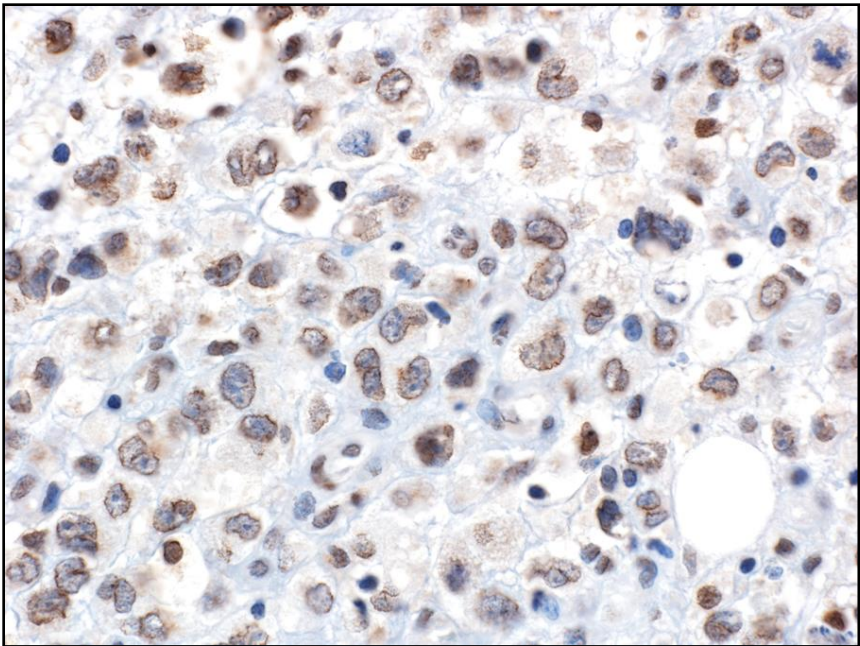

C

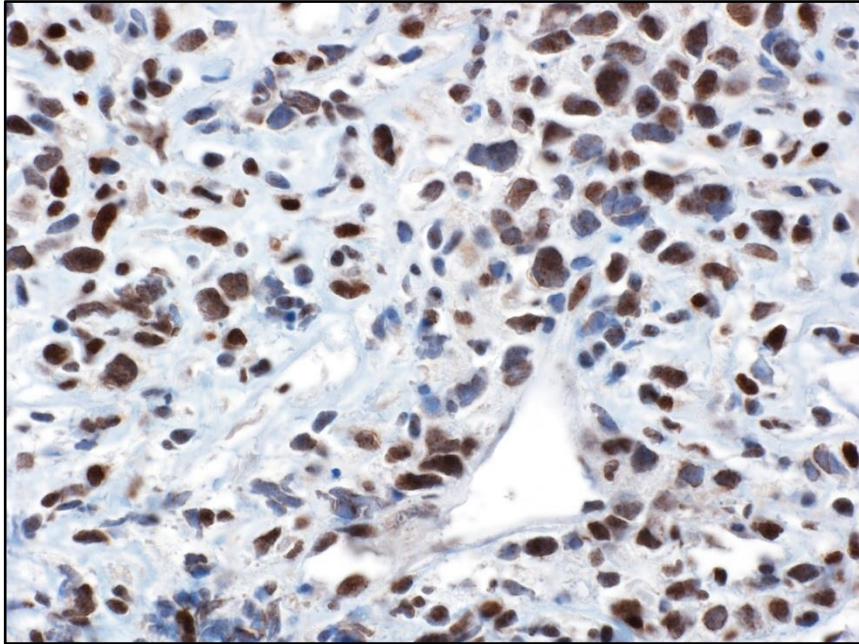

Supplement: Supplementary file 2 — Supplemental Figure 1 [file 41408_2019_188_MOESM2_ESM.pdf]

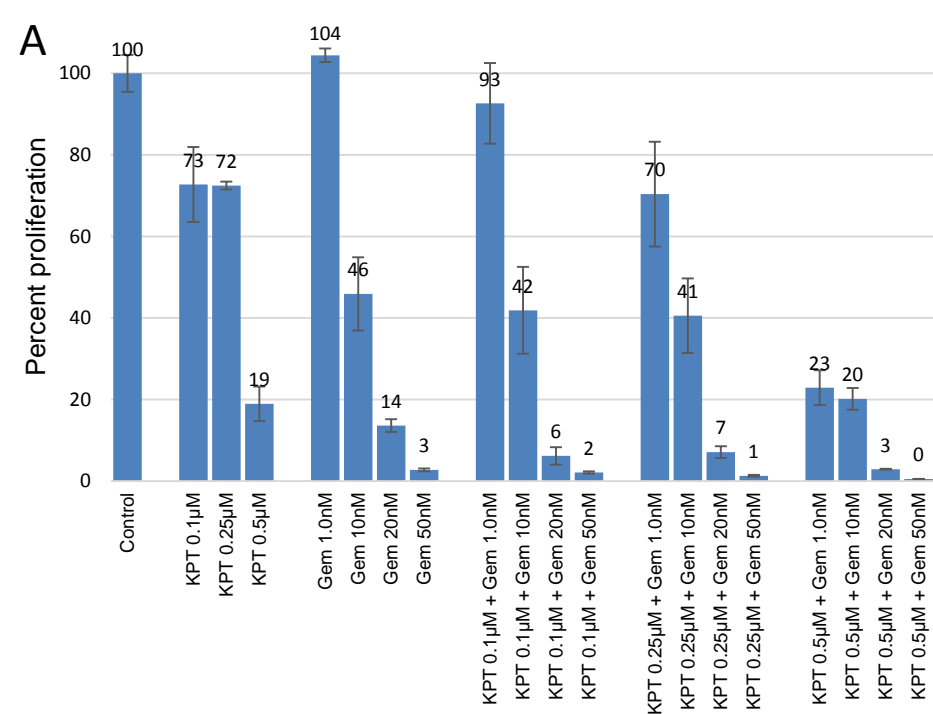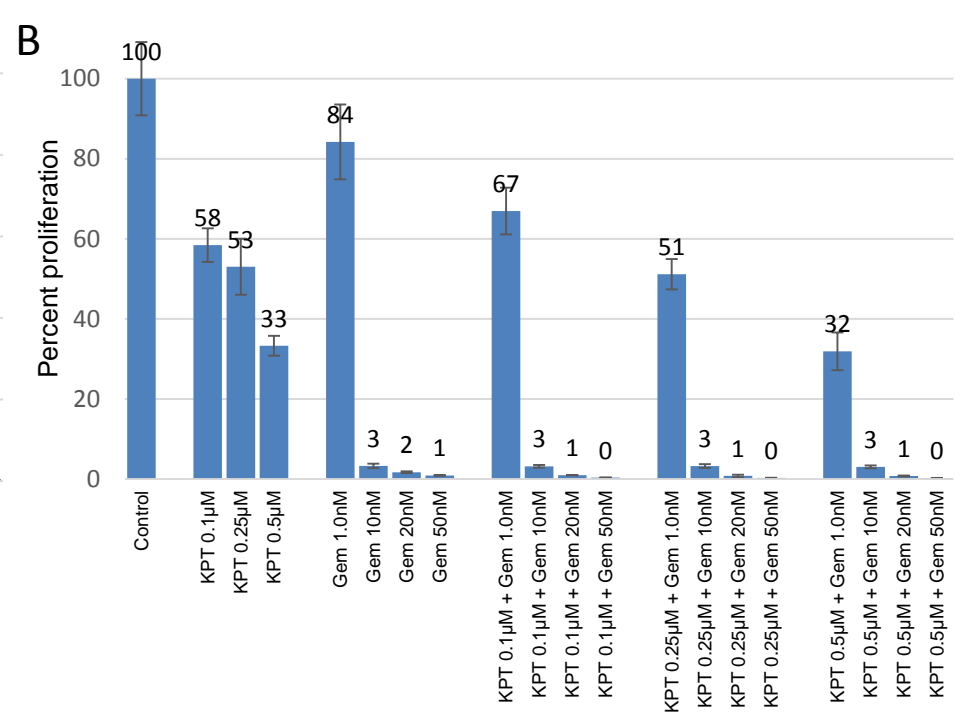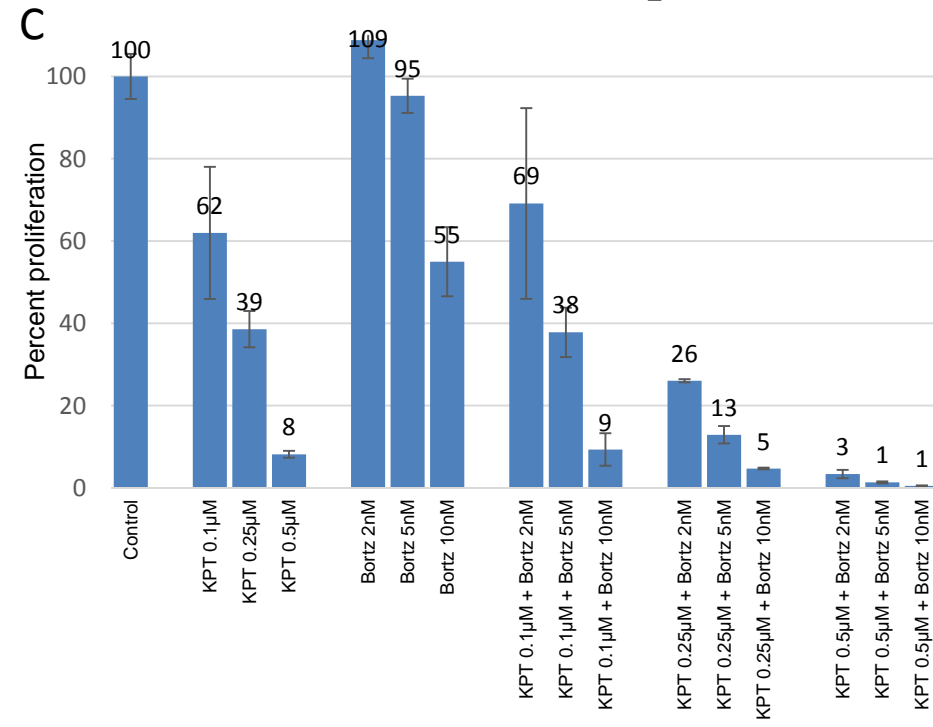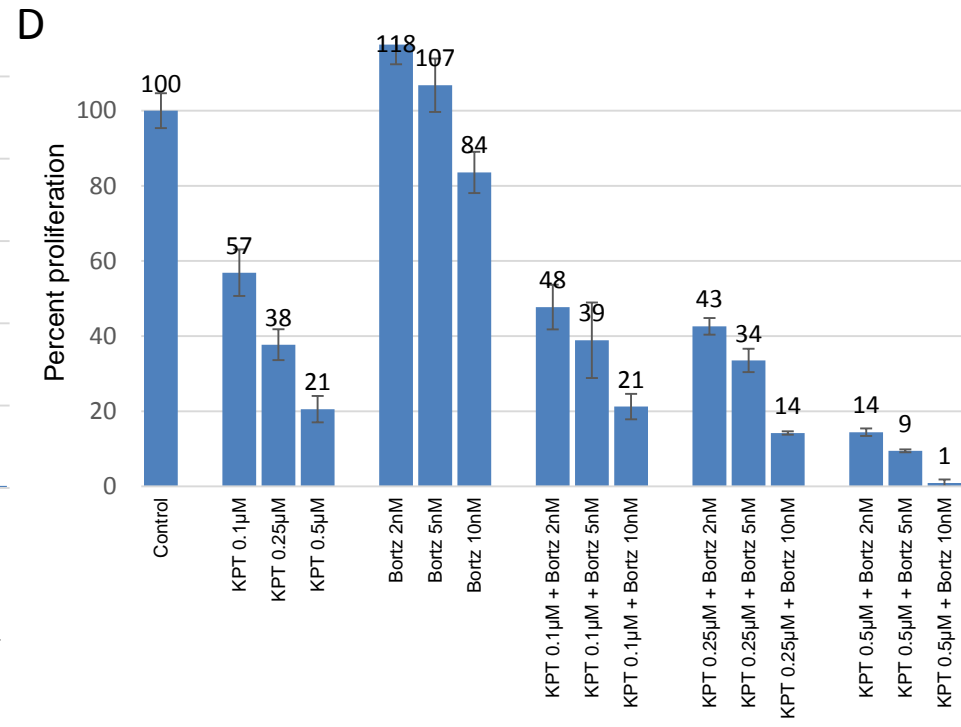

Supplement: Supplementary file 3 — Supplemental Figure 2 [file 41408_2019_188_MOESM3_ESM.pdf]
